# Stimulus-Dependent State Transition between Synchronized Oscillation and Randomly Repetitive Burst in a Model Cerebellar Granular Layer

**DOI:** 10.1371/journal.pcbi.1002087

**Published:** 2011-07-14

**Authors:** Takeru Honda, Tadashi Yamazaki, Shigeru Tanaka, Soichi Nagao, Tetsuro Nishino

**Affiliations:** 1 Department of Information and Communication Engineering, Graduate School of Electro-Communications, The University of Electro-Communications, Chofu-shi, Tokyo, Japan; 2 Laboratory for Motor Learning Control, RIKEN Brain Science Institute, Wako-shi, Saitama, Japan; 3 Strategic Planning Unit, RIKEN BSI-TOYOTA Collaboration Center, RIKEN Brain Science Institute, Wako-shi, Saitama, Japan; 4 Department of Informatics, Graduate School of Informatics and Engineering, The University of Electro-Communications, Chofu-shi, Tokyo, Japan; University of Freiburg, Germany

## Abstract

Information processing of the cerebellar granular layer composed of granule and Golgi cells is regarded as an important first step toward the cerebellar computation. Our previous theoretical studies have shown that granule cells can exhibit random alternation between burst and silent modes, which provides a basis of population representation of the passage-of-time (POT) from the onset of external input stimuli. On the other hand, another computational study has reported that granule cells can exhibit synchronized oscillation of activity, as consistent with observed oscillation in local field potential recorded from the granular layer while animals keep still. Here we have a question of whether an identical network model can explain these distinct dynamics. In the present study, we carried out computer simulations based on a spiking network model of the granular layer varying two parameters: the strength of a current injected to granule cells and the concentration of Mg^2+^ which controls the conductance of NMDA channels assumed on the Golgi cell dendrites. The simulations showed that cells in the granular layer can switch activity states between synchronized oscillation and random burst-silent alternation depending on the two parameters. For higher Mg^2+^ concentration and a weaker injected current, granule and Golgi cells elicited spikes synchronously (synchronized oscillation state). In contrast, for lower Mg^2+^ concentration and a stronger injected current, those cells showed the random burst-silent alternation (POT-representing state). It is suggested that NMDA channels on the Golgi cell dendrites play an important role for determining how the granular layer works in response to external input.

## Author Summary

Intensive studies of Pavlovian delay eyelid conditioning suggest that the cerebellum can memorize a passage-of-time (POT) from the onset of an external stimulus. To account for possible mechanisms of such POT representation, some network models have been proposed to show that granule cells (grcs) in the cerebellar granular layer can exhibit random alternation of burst and silent modes under feedback inhibition from Golgi cells, resulting in non-recurrent generation of active granule cells populations. On the other hand, the oscillation of local field potential (LFP) has been observed in the cerebellar granular layer when animals stay at rest. Some network models have shown that grcs can elicit synchronous spikes in an oscillatory manner. These qualitatively different neural dynamics of the granular layer raises a question of how they can be accounted for by an identical network in the granular layer. Here we report that grc activities of a biologically plausible spiking network model undergo the state transition between synchronized oscillation and random burst-silent alternation, depending on the activation of NMDA channels on the Golgi cell dendrites and the strength of a current injected to grcs.

## Introduction

The cerebellar granular layer is one of the stations receiving external stimuli for information processing of the cerebellar cortex. The granular layer is thought to transform spatial patterns of mossy fibers (MFs) input signals into a population of active granule cells (grcs) [1], [2]. Recently, Yamazaki and Tanaka [3] have proposed that the granular layer transforms spatiotemporal patterns of MF input signals into a sparse population of active grcs in the presence of inhibitory Golgi cells (Gocs), and suggested that the passage of time (POT) from the onset of MF signals is represented by the granular layer network.

On the other hand, when an animal stays at rest without any external stimuli, oscillatory local field potential (LFP) is observed in the granular layer at 7–8 Hz in rats [4] and at 13–14 Hz in monkeys [5], [6]. Although the origin of this oscillation remains unknown, Maex and De Schutter [7] proposed that grcs and Gocs become active alternatingly and repeatedly by the recurrent connections, and their average oscillatory firing may be observed as the oscillation of LFP.

There are two distinct dynamics in the cerebellar granular layer: one for the POT representation and the other for the synchronized oscillation. Assuming that the two dynamics emerge from the same neural circuit, one may expect that the strength of external input controls the transition between the two dynamics. However, models accounting for the POT representation do not exhibit the synchronized oscillatory firing of grcs and Gocs but generate spikes randomly for weak external input [3], whereas models accounting for the synchronized oscillation persistently show the oscillatory state even for strong external input [7]. Therefore, it is not trivial to explain the possibility that the two dynamics take place in the same model of the cerebellar granular layer. In this study, we demonstrate that a spiking network model of the cerebellar granular layer can generate synchronized oscillation for weak external input and active grc populations representing the POT for strong external input.

## Materials and Methods

### Overview of the present model

**Figure 1:**
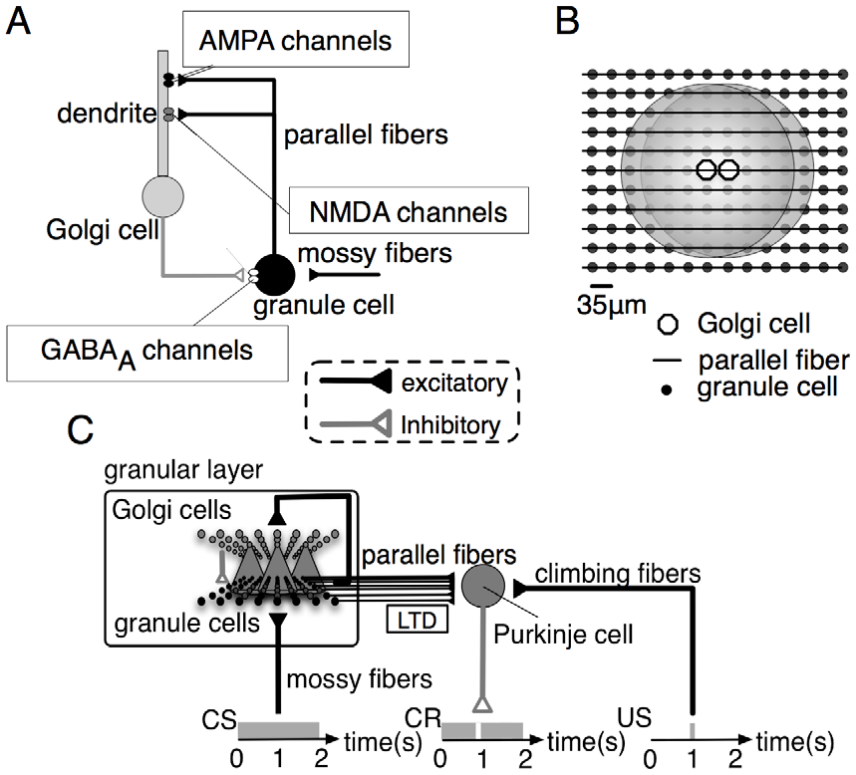
Schematic diagrams of the present cerebellar model and external input stimuli in delay eyelid conditioning. ***A***, A local circuit composed of a granule cell, a Golgi cell and mossy fibers with synaptic channels. AMPA and NMDA channels are assumed to exist on the Golgi cell dendrites, and GABA_A_ channels are assumed to exist on the granule cell. ***B***, 2-dimensional arrangement of granule cells, Golgi cells and parallel fibers in the model granular layer. Small dots and open circles indicate granule cells and Golgi cells, respectively. Horizontal lines indicate parallel fibers, which terminate on the Golgi cell dendrites shown by gray disks. ***C***, During delay eyelid conditioning, an animal receives the repeated paired presentation of a sustained tonal conditioned stimulus (CS) and an airpuff that is a delayed unconditioned stimulus (US) inducing an eyeblink response. Mossy fibers convey CS signals to granule cells, which send excitation signals to Purkinje cells through parallel fibers, whereas climbing fibers convey US signals to Purkinje cells. Synaptic weights between parallel fibers and Purkinje cells undergo long-term depression (LTD) when the parallel fibers excite Purkinje cells at the timing of the signal input through climbing fibers. After conditioning, the animal elicits an eyeblink as a conditioned response (CR) to a CS around the timing of the airpuff.

We build our model on the basis of the GENESIS script of the granular layer model for synchronized oscillation, which was written by Maex and De Schutter [7]. Briefly, we extend the original single-compartment Goc model to a multi-compartment model composed of a soma and a dendrite, on which *N*-methyl-*D*-aspartate (NMDA) channels are distributed (Fig. 1***A***). We also extend their one-dimensional network structure to a two-dimensional one and set random sparse connections between the model Gocs to the grcs (Figs. 1***B*** and ***C***). We modified the values of the time constant and conductance of GABA_A_ channels on a grc soma, and that of the conductance of AMPA channels on a Goc dendrite (see below and Table S1) so that simulation reproduces the synchronized oscillation reported by Maex and De Schutter [7].

### Network structure

We build a model granular layer of the cerebellum using the network structure employed in our previous study [3]. That is, 32×32 model Gocs are arranged in two-dimensional grids (Figs. 1***B*** and ***C***), which is in contrast with the one-dimensional arrangements of model Gocs and grcs in the study of Maex and De Schutter [7]. Our model Gocs are evenly positioned at 35 µm intervals within a square sheet of 1,085×1,085 µm^2^. It was estimated that there are 1,000 times more grcs than Gocs [8], [9]. Numerous grcs are connected with a glomerulus [10]. However, simulation with more than 1 million model neurons is beyond the power of our computers. In Yamazaki and Tanaka [3], 100 nearby grcs that were assumed to be connected with a glomerulus exhibited similar firing patterns despite each of the grcs independently received noisy signals through MFs. Such redundant activity patterns of grcs suggest that a large number of grcs behave as a single cluster when they receive inputs from the nearest Goc through a single glomerulus. In the present model, for the sake of the economy of computer power, we assume that a single model grc represents a grc cluster composed of about 1,000 neurons.

We also assume that a model Goc randomly receives 10% of 9×32 PF inputs from model grcs with their dendritic arborization, whose diameter is set at 315 µm. The mean and standard deviation of the actual number of PF inputs to a model Goc were 26.80 and 6.50, respectively. The model Goc, in turn, sends inhibitory inputs to model grcs located within the extent of axonal arborization (Fig. 1***B***), which is set at 315μm. The number of model Gocs located inside a circle of the diameter of 315μm amounts to 69 by actual counting, which can be roughly estimated by *π*(315/2)^2^/(35-1)^2^+1 = 68.4. In the present model we assume that each grc randomly receives 10% of 69 connections from Gocs. The mean and standard deviation of the actual number of connections were 6.11 and 2.57, respectively. For simplicity, we omit MF inputs to model Gocs.

### Model granule cells

We use the same model grcs as those adopted by Maex and De Schutter [7] except for the synaptic channels. Brickley et al. [11] reported that inhibitory postsynaptic potentials (IPSPs) can be fitted well with the sum of three exponentials, and that the largest component, which has the decay time constant of 100 ms, contributes to 20% of the IPSPs. Considering this finding, we simulate IPSPs using a double-exponential function with rise and decay time constants of 5 and 100 ms, respectively [11]. Further details are shown in Table S1.

### Model Golgi cells

In Maex and De Schutter [7], a model Goc was also a single-compartment Hodgkin-Huxley unit with realistic ion channels. Their model Gocs received excitatory inputs from model grcs through α-amino-3-hydroxy-5-methyl-4-isoxazolepropionic acid (AMPA) channels with rise and decay time constants of 0.03 and 0.5 ms, respectively [12].

It has been found that Gocs receive excitatory input signals from PFs through not only AMPA channels but also NMDA channels [13]. However, even when we add NMDA channels on the somas of model Gocs, the NMDA channels do not open effectively to evoke sustained depolarization because the after-hyperpolarization (AHP) [14], [15] following the generation of each action potential rapidly decreases the somatic membrane potential. On the other hand, it is known that the dendritic potential tends not to be affected by AHP at cell somas [14]. Moreover, it has been shown that direct dendritic excitation produces sustained and burst responses although somatic excitation does not [14]. If NMDA channels really induce a prolonged activation of Gocs, it implies that NMDA channels are located on the Goc dendrites. In this study, a model Goc is represented as a soma and a dendrite whose length is 300 µm (cf. [16]) (Fig. 1***A***). The dendrites of model Gocs are assumed to possess AMPA and NMDA channels. We simulate NMDA receptor (NMDAR)-mediated excitatory postsynaptic potentials (EPSPs) with a double-exponential function with rise and decay time constants of 5 and 100 ms, respectively, in accordance with Misra et al. [17]. Further details are shown in Table S1.

It is known that Gocs are morphologically heterogeneous and their action potentials are variable [18]. Although it is likely that random connectivity in the network would have smeared individual heterogeneities, a reversal potential for the leak current at model Gocs is distributed uniformly between −60 and -50 mV according to Maex and De Schutter [7].

### Stimulus and simulation paradigm

We model MF input signals as a current injected directly to the soma of each model grc, instead of feeding spike trains. Freeman and Muckler [19] have reported that the spontaneous firing rate of MFs is as low as 5 Hz, whereas the firing rate increases up to 30 Hz when an animal is stimulated with a tone. We assume that the influx of a current to grcs increases with the frequency of firing conveyed through MFs. In the simulation, we inject a current *I*_MF_ of 10.7 pA to all model grcs for 2 s. For the succeeding 2 s, we inject a current *I*_MF_ of 22.7 pA to evoke activity in grcs. Subsequently, we inject a current *I*_MF_ of 10.7 pA for 2 s again to resume the baseline activity of grcs. In total, we simulate the dynamics of the granular layer network for 6 s. Note that we omit noise in the injected current.

### Simulation tools

We build the present model and perform numerical simulations using the GENESIS simulator [20]. Differential equations are solved using the Crank-Nicolson method with a fixed time step of 20 µs. When we carry out simulation of eyelid conditioning, we do not need to calculate activities of the granular layer at the same time. We have only to use activity patterns of grcs, which have been stored in a file recorded during the simulation of activities of the granular layer using GENESIS. Because we assume a simple PC model, the simulation does not require the usage of GENESIS that is an expert for simulation of realistic neuron models. Therefore, the simulation program of the model Purkinje cell (PC) for eyelid conditioning, which is just solving differential equations using the first order Euler method with a fixed time step of 20μs, was written in C and C++ programming languages.

### Analyses of simulated results

Let 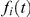 be the spike activity of model neuron (grc or Goc) *i* (1≤*i*≤*N*, *N* = 32×32 = 1024) at time *t*:
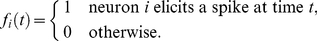
(1)The fluctuation in the number of neurons that elicit spikes at time *t*, 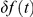, is given by
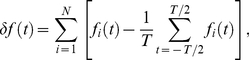
(2)The normalized autocorrelation function of the numbers of active neurons at times *t* and *t* + *τ* is defined by
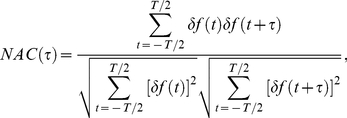
(3)where *τ* is a time lag and *T* is the duration of weak or strong external input. Calculating the normalized Fourier cosine transform of this normalized autocorrelation function, we define the oscillation index *OI*, which measures the degree of synchronized oscillation at neurons as follows:
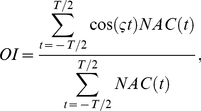
(4)where 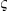 is the frequency giving a maximum power defined by the Fourier cosine transform of the normalized autocorrelation function. When *OI* takes a value of 1, a population of active neurons appears periodically. When *OI* takes a value of 0, *NAC*(*τ*) is a constant function of *τ*, and populations of active neurons appear uniformly in time.


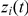
 is defined as the AMPA receptor (APMAR)-mediated EPSPs on a PC from grc *i*, as follows:
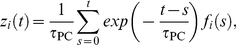
(5)where 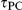 is the decay time constant of AMPAR-mediated EPSPs on a PC, which is set at 30 ms. As defined in the previous study [3], we use the similarity index 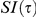 of the function of *τ*, which is given by
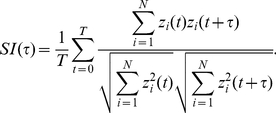
(6)The right-hand side of this equation represents the extent to which two populations of active neurons at different times with interval *τ* are correlated. If 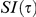 takes a value of 1, the populations at times *t* and *t* + *τ* are identical, and when 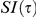 takes a value of 0, different populations are active at times *t* and *t* + *τ*. The monotonic decrease in 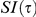 with increasing |*τ*| indicates that the population of active neurons changes with time without recurrence [21]. In addition, we also define another index that measures the ability of POT representation. The ideal POT representation is achieved by monotonically decreasing 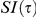 with respect to |*τ*| with a large difference between 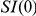 and the minimum of 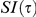 [3]. However, even in the cases of oscillatory activities overlaid on the random repetition of burst and silent activities, if the temporal coherence of the oscillation decreases with increasing *τ*, the POT representation is possible to some extent. To extract a residual POT-representing component of the active neurons' population, we first fit 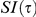 with a Gaussian function and calculated the height between 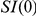 and the minimum of 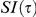. The height is then divided by 0.376, which is the largest value of the height among all similarity indices examined by a brute-force search varying Mg^2+^ concentration ([Mg^2+^]) and input current strength. We call this normalized height the POT-representation index (*PI*).

To examine the reproducibility of active neurons' population among different trials of the same external input, we use the previously introduced reproducibility index 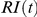 [3], [21], given by
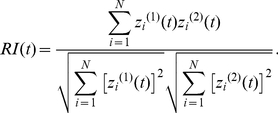
(7)Here, the superscripts (1) and (2) indicate two different trials. 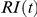 represents the similarity between the populations of active neurons at time *t* measured from the onset of injection of a current to grcs in two different trials. When 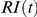 takes a value of 1, the populations of active neurons in the two trials are identical, and when it takes a value of 0, they are completely different [3].

### Simulation of delay eyelid conditioning

In order to confirm that the POT-representing state generated by our model functionally serves for inter-stimulus interval representation in Pavlovian delay eyelid conditioning [22]–[24], we conduct simulations assuming that strong current injection to grcs corresponds to a neural signal of a conditioned stimulus (CS) conveyed through MFs and a neural signal from the inferior olive through climbing fibers corresponds to an unconditioned stimulus (US) (Fig. 1***C***). The sustained CS is fed to grcs, whereas the US is sent to PCs with a certain delay. We assume that a large current injection to all grcs corresponds to a CS presentation, and a small current injection corresponds to the input of spontaneous MF activity. We employ a Hodgkin-Huxley unit as a model PC assuming that the PC receives excitatory inputs from all Parallel fibers (PFs) (Fig. 1***C***). PF input signals are modeled as AMPAR-mediated EPSPs. The decay time constant is set at 30 ms [25], and the peak conductance is set so that the model PC elicits spikes at a maximum rate of 100 spikes/s in response to a CS [26]. We assume that the US is fed either 0.5, 0.75 or 1.0 s after the CS onset and that simulated long-term depression (LTD) is induced when PFs and a climbing fiber are coactivated within a brief time window [16]. We set initial synaptic weights of PF*_i_* in the model PC, *w_i_* ^(0)^, to 1. When grc *i* fires 0.05–0.1 s before the onset of a US, *w_i_* is set to 0; otherwise, *w_i_* is not changed. For simplicity, we assume that US signals contribute to the induction of LTD at PFs, and we do not take into account their contributions to the dynamics of the PC activity in simulations.

## Results

### Input-dependent transition of dynamical states in the model granular layer

**Figure 2:**
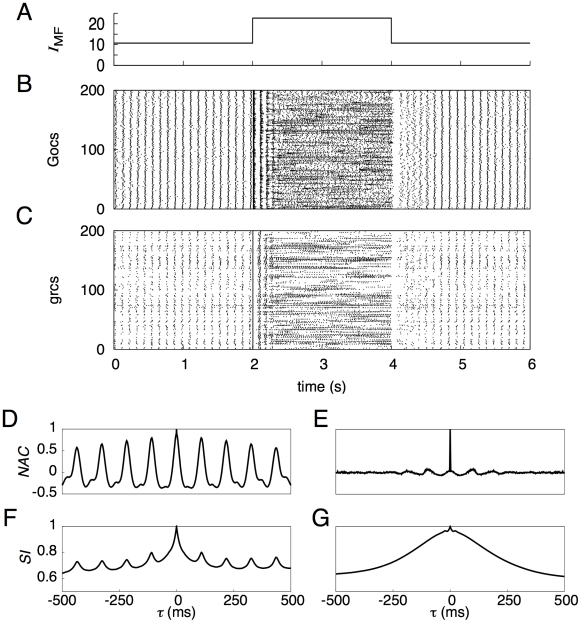
Activities of grcs and Gocs in response to a current injected to model grcs. ***A***, Temporal pattern of an injected current, in which a small current was injected for 0–2 and 4–6 s, whereas a large current was injected for 2–4 s. ***B*** and ***C***, Spike patterns of 200 out of 1,024 grcs and 200 Gocs, respectively. During the injection of a small current, grcs and Gocs elicited spikes synchronously and rhythmically. During the injection of a large current, the populations of active grcs and Gocs exhibited random alternation between the burst and silent modes. ***D*** and ***E***, Normalized autocorrelation functions, *NAC*(*τ*), during the injection of a small and a large currents, respectively. ***F*** and ***G***, Similarity index functions, 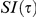, of grcs are shown during small and large current injections, respectively. *NAC*(*τ*) indicates the oscillatory generation of spikes and 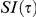 shows that similar spike patterns were repeatedly generated, although the spike patterns gradually became different gradually with the separation of time during the small current injection. On the other hand, during the large current injection, *NAC*(τ) indicates the random spike generation and 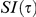 shows that the spike pattern changed gradually and that each pattern was only generated once.

Figures 2***B ***and ***C*** represent the temporal patterns of spikes generated by 200 representative model Gocs and grcs, respectively, in response to the injection of a current to the grcs (Fig. 2***A***). For the first 2 s, in which a small current was fed to the grcs, simulating a response to spontaneous MF activity, model grcs and Gocs elicited spikes rhythmically and synchronously with a frequency of 9 Hz. The emergence of the synchronized oscillation in the grcs and Gocs is consistent with the findings of Maex and De Schutter [7]. For the successive 2 s, in which a large current was injected to the grcs, the model grcs exhibited random alternation between burst and silent modes, consistent with the findings of Yamazaki and Tanaka [3]. After the current amplitude was decreased to that for the first 2 s, the network quickly returned to the oscillatory state at 9 Hz. These simulation results show that the model cerebellar granular layer can exhibit two qualitatively distinct dynamical states depending on the strength of the MF input signal. In the following subsections, we examine the properties of each dynamical state in detail.

### Synchronized oscillation generated by a small current injection

To confirm the generation of synchronized oscillation at model grcs in response to the injection of a small current (0–2 s), we calculated a normalized autocorrelation function *NAC*(*τ*) of the activities of the grcs using Equation 3 and the value of the oscillation index *OI* using Equation 4. We found a clear oscillation at 9 Hz, as shown in Fig. 2***D***. We also obtained *OI* = 0.836, suggesting robust synchronization of the activities of the model grcs. This oscillation frequency was in the range of frequencies of oscillatory LFP observed in the cerebellar cortex by Hartmann and Bower [4] and Pellerin and Lamarre [5] (7–8 Hz in rats and 13–14 Hz in monkeys). This frequency was lower than that shown by Maex and De Schutter [7] due to a larger decay constant of γ-aminobutyric acid-A receptor (GABA_A_R)-mediated IPSPs on model grcs (0.31 ms (rise) and 8.8 ms (decay) for Maex and De Schutter [7]; 5.0 ms (rise) and 100 ms (decay) for the present model). These results suggest that the granular layer of the biological cerebellum is in a state of synchronized oscillation under spontaneous MF signal input. The similarity between spike activity patterns at different times decreased with the time interval, but patterns showed weak oscillation associated with synchronized oscillation (Fig. 2***F***). The *NAC*(*τ*) and similarity index 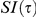 for model Gocs were similar to those for model grcs (Fig. S1).

The emergence of synchronized oscillation may be interpreted in terms of the following circuit mechanism between grcs and Gocs. The relatively low average firing rate of grcs induced by a small input current evokes the weak depolarization of Gocs, which rarely opens voltage-gated NMDA channels on the Goc dendrites. Thereby, the Gocs elicit spikes only sparsely followed by a refractory period. The low-frequency spike activity of the Gocs inhibits grcs, and the grcs become inactive. After the recovery from the inhibition from the Gocs, the grcs become active due to the sustained external current input and elicit low frequency spikes again. The repetition of such activation and deactivation processes results in the synchronized oscillation of the model grcs and Gocs. This mechanism for the emergence of the oscillatory state is common to that reported by Maex and De Schutter [7].

The average firing rate of individual grcs was less than half (≈4 spikes/s) of the oscillation frequency (9 Hz) in our simulations. This indicates that not all grcs were activated at every oscillation cycle, which is attributed to the random and sparse connectivity between the Gocs and grcs. When the inhibition of the grcs by the Gocs was removed, the grc firing rate increased to 44 spikes/s. This result suggests that the synchronized oscillation was generated by the network dynamics, rather than the intrinsic mechanisms of individual grcs under the constant current injection.

### Random burst-silent alternation generated by a large current injection

Figures 2***B*** and ***C*** also represent spike patterns of 200 model Gocs and grcs, respectively, in response to the injection of a large current to the grcs in the interval from 2 to 4 s. Model grcs exhibited random alternation between burst and silent modes. The normalized autocorrelation *NAC*(*τ*) is markedly reduced except at *τ* = 0 ms (Fig. 2***E***). Different grcs showed different patterns of spike trains. To determine whether the same active grc populations appear more than once, we calculated the similarity index 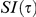 using Equation 6 and plotted it in Fig. 2***G***. The value of 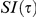 was 1 at *τ* = 0 ms because of the trivial identity. 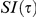 monotonically decreased as |*τ*| increased with the POT-representation index *PI* = 0.923. This indicates that the population of active grcs changed gradually with time from the onset of a large current injection without the recurrence of active grc populations, as reported by Yamazaki and Tanaka [3]. The *NAC*(*τ*) and 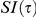 for model Gocs were similar to those for model grcs (Fig. S1). To determine the effect of random connections between model grcs and Gocs, we performed simulations using a modified model in which grcs and Gocs were all connected with each other so that the network did not have any randomness. As a result, activities of these cells exhibited only coherent oscillations (data not shown). This suggests that the random connectivity is a major cause of generating the random alternation between burst and silent modes in grcs and Gocs.

The random alternation between burst and silent modes of grcs can be accounted for by the following mechanism. The strong activation of grcs by the large current injection vigorously depolarizes randomly connected Gocs, resulting in the activation of voltage-gated NMDA channels on the dendrites of the Gocs. Because of the long decay time constant of NMDAR-mediated EPSPs, the Gocs send sustained inhibitory signals to nearby grcs, so that the grcs become inactive. Then a Goc that receives inputs from these grcs decreases its activity, which reactivates the grcs. However, due to the random connections, the timings of the reactivation and deactivation of grcs are different for different grcs, resulting in the random alternation between burst and silent modes of grcs.

### Reproducibility of active grc populations in POT-representing states

**Figure 3:**
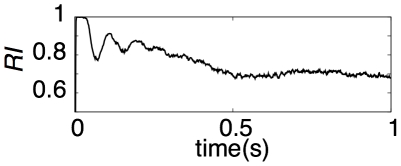
Reproducibility of spike patterns of grcs for injection of a large current.

To confirm that the present model achieves reproducible POT representation for different trials of the strong current injection, we carried out the following simulations. We injected a large current to grcs twice during 2–4 and 6–8 s, whereas we injected a small current at other times. We evaluated the reproducibility index 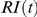 between active grc populations in response to the first and second injections of a large current using Equation 7 (Fig. 3). 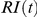 immediately after the onset of the current injection (*t*<50 ms) was 1, indicating a perfect reproducibility. Afterward, it tended to decrease with the duration of the current injection. The perfect reproducibility observed around the onset of the current injection originates in the simultaneous activation of all grcs at the onset followed by strong inhibition from Gocs. This resetting mechanism enables the network to lose its history of activities. Consequently, the present model can represent the POT robustly and reproducibly for different trials of a large current injection.

### Network dynamics at high Mg^2+^ concentration

To confirm the importance of NMDA channels assumed on the Goc dendrites for the network dynamics, we blocked the channels by increasing the concentration of Mg^2+^ (1.20 mM → 13.0 mM) and carried out a simulation.

**Figure 4:**
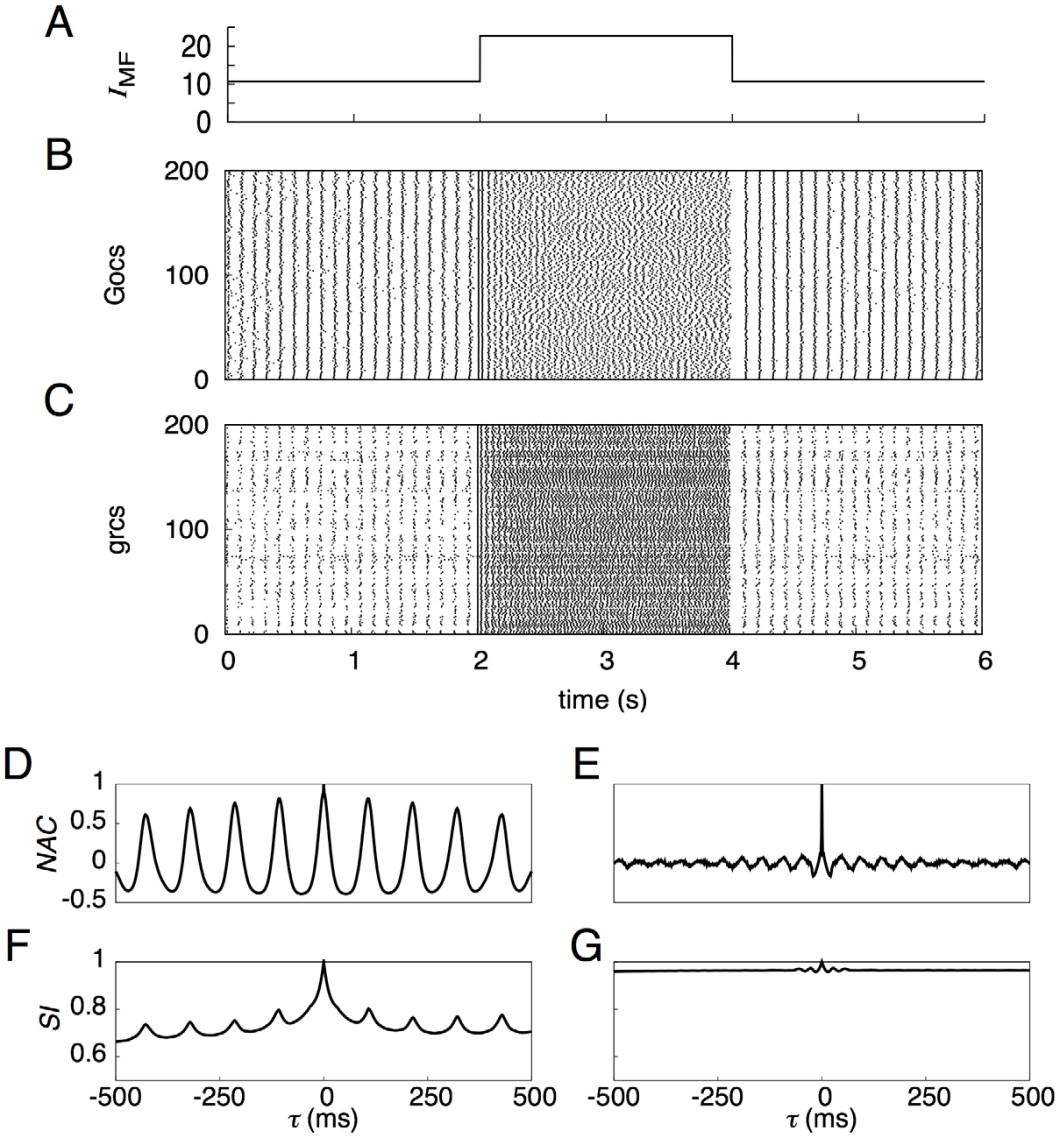
Activities of model grcs and Gocs when NMDA channels of model Gocs were blocked. Conventions are as in Fig. 2. ***B*** and ***C*** show that the grcs and Gocs elicited spikes synchronously and rhythmically during the small current injection, and that they generated spikes randomly during the large current injection. Even for the blockade of NMDA channels, the statistical features of the activity patterns of grcs during the small current injection (***D*** and ***F***) were the same as those shown in Figs. 2***D*** and ***F***. When a large current was injected, the sufficiently small amplitude of oscillation in *NAC*(*τ*) shown in ***E*** indicates that grcs elicited spikes randomly, and the constant 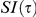 shown in ***G*** indicates that active grc populations were almost the same.

Figure 4 shows spike trains elicited by 200 Gocs (Fig. 4***B***) and 200 grcs (Fig. 4***C***) in response to a current injection (Fig. 4***A***) under the blockade of the NMDA channels on the model Gocs. The synchronized oscillation of grcs and Gocs was unaffected during the injection of a small current to the grcs for 0–2 and 4–6 s. This was observed by the appearance of oscillatory behavior of the normalized autocorrelation function *NAC*(*τ*) (Fig. 4***D***). The similarity index 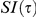 resembled that in the default case (Fig. 4***F***). As mentioned previously, when the injected current was small, the NMDA channels on the Goc dendrites rarely opened, irrespective of whether NMDA channels were blocked. Therefore, the oscillatory behavior of grcs and Gocs during small current injections was preserved under the blockade of NMDA channels. On the other hand, in response to a large current injection to the grcs for 2–4 s, the grcs elicited spikes at a higher firing rate than in the default case (13 spikes/s → 32 spikes/s) and the spike patterns were random and uniform, as shown in Fig. 4***C***. This observation was justified by the fact that *NAC*(*τ*) showed oscillation whose amplitude was sufficiently small compared with its value at *τ* = 0 (Fig. 4***E***). The grcs did not show random burst-silent alternation. This was caused by the weaker inhibition from Gocs due to the blockade of NMDA channels. As shown in Fig. 4***G***, 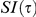 became much higher than in the default case. The POT-representation index *PI* was as small as 0.009, compared with the value of 0.923 in the default case. This indicates that the temporal sequence of active grc populations does not represent the POT even during a large current injection. We confirmed a similar tendency for the activity patterns of model Gocs (Fig. S1).

Taken together, the activation of NMDARs on the Goc dendrites is not important for the generation of synchronized oscillation of grc and Goc activities. In contrast, the activation of the NMDARs is indispensable for the generation of random burst-silent alternation, which enables the network to represent the POT.

### Network dynamics at low Mg^2+^ concentration

Next, to examine whether the oscillatory states are affected by the reduced Mg^2+^ concentration, we performed a simulation under a nearly [Mg^2+^]-free condition (0.013 mM).

**Figure 5:**
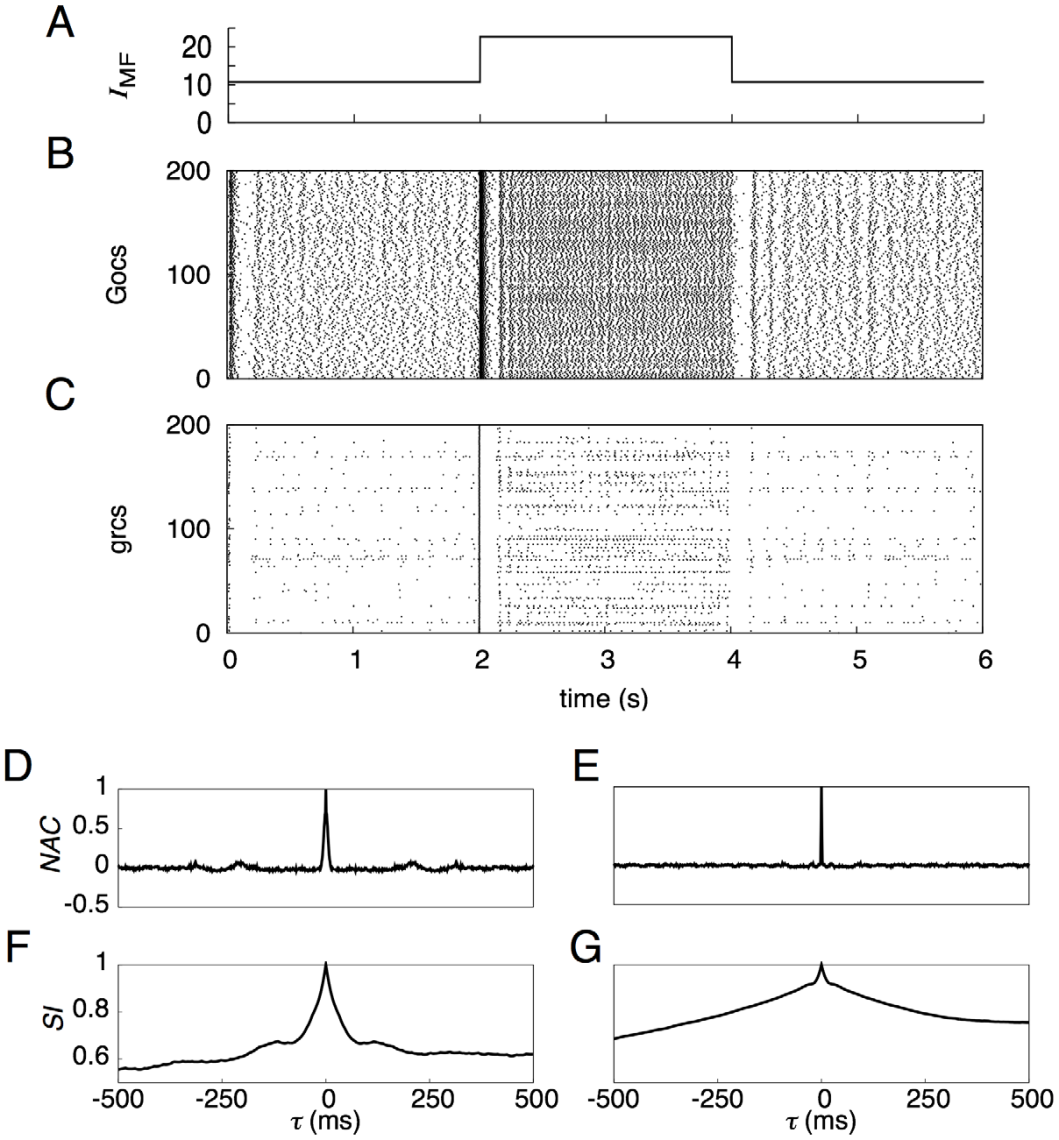
Activities of grcs and Gocs when NMDA channels of Gocs were continually open. Conventions are as in Fig. 2. When a small current was injected, the Gocs elicited spikes at high frequencies (***B***), which in turn strongly inhibited the grcs. Consequently, the generation of oscillatory spikes at grcs disappeared (***C***). The absence of the oscillation was demonstrated by the sharp peak of *NAC*(*τ*) at τ = 0 (***D***). The overlap of active grc populations decreased rapidly with the separation of time (***F***). When a large current was injected, the Gocs also fired at high frequencies, too (***B***). In this case, however, the populations of active grcs exhibited random alternation between the burst and silent modes although the grcs elicited spikes sparsely (***C***, compared with Fig. 2***C***). This random spike pattern was demonstrated by the sharply localized peak of *NAC*(*τ*) at *τ* = 0 and otherwise, *NAC*(*τ*) ≈0, as shown in ***E***. 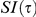 monotonically decreased as |τ| increased (***G***), indicating that the population of active grcs changed gradually to different populations with time without recurrence.

Figures 5***B*** and ***C*** show spike trains of 200 Gocs and grcs, respectively, in response to the current injection to the grcs (Fig. 5***A***). The spike trains of the grcs exhibited random burst-silent alternation in response to a large current injection for 2–4 s. The observation of randomness in the spike activity patterns was justified by the fact that the normalized autocorrelation function *NAC*(*τ*) took almost zero except at *τ* = 0 (Fig. 5***E***). Although the firing rate of the grcs was reduced by the enhanced inhibition from the Gocs, the similarity index 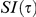 decreased monotonically with *τ* (Fig. 5***G***), as observed in the case where the POT is represented. On the other hand, for 0–2 and 4–6 s, during which a small current was injected, the grcs and Gocs did not undergo synchronized oscillation (Fig. 5***D***). The grcs generated spikes sparsely in a stochastic manner (the oscillation index *OI* = 0.207), whereas the Gocs elicited spikes at almost the same frequency as that in the default setting. 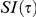 monotonically decreased, reflecting the absence of synchronized oscillation (Fig. 5***F***). These results indicate that the persistent reduction of Mg^2+^ concentration disrupts the synchronized oscillation. We confirmed a similar tendency for the activity patterns of model Gocs (Fig. S1).

### State transition controlled by Mg^2+^ concentration

Here we examine how the synchronized oscillation and POT-representing states emerge depending on the Mg^2+^ concentration.

**Figure 6:**
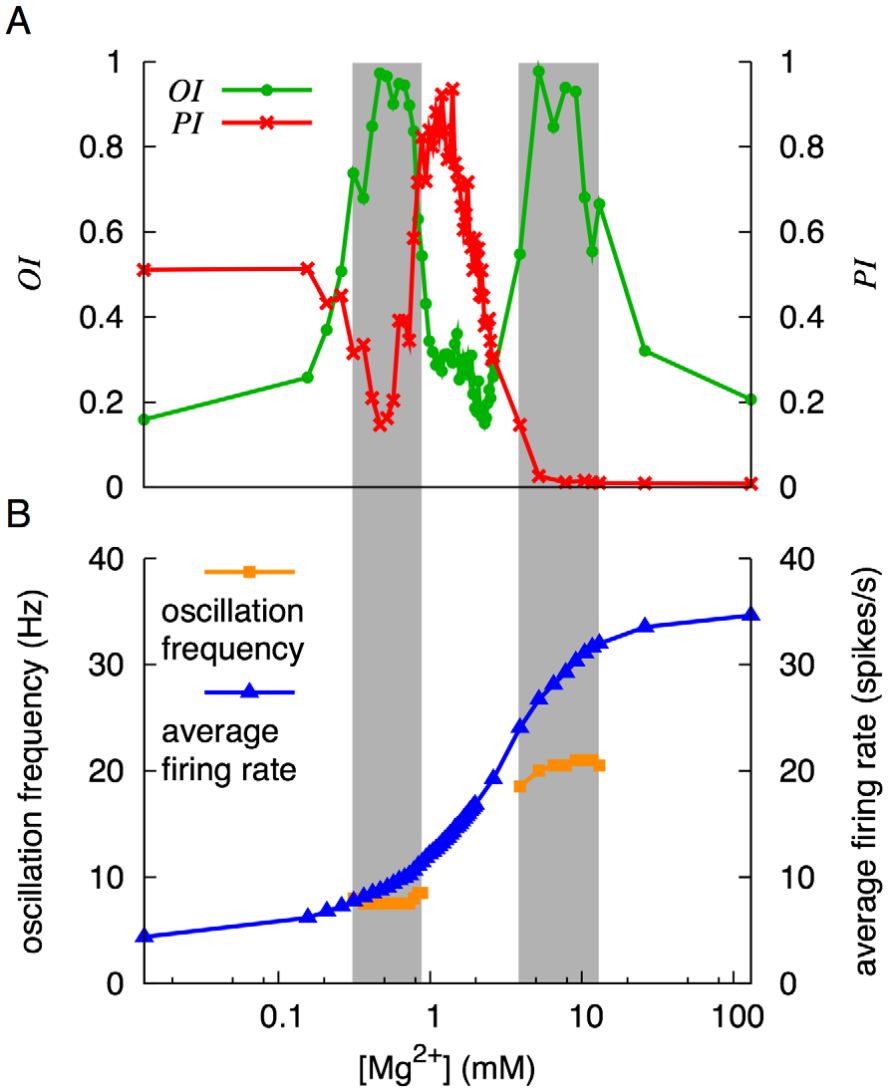
Dynamical states of the network during a current injection (*I*_MF_ = 22.7 pA) with various concentrations of Mg^2+^. ***A***, Dependence of the oscillation index (*OI*) and POT representation index (*PI*) on the concentration of Mg^2+^. *OI* was large in two separate regions of Mg^2+^ concentration, which are indicated by the vertical gray bands. In-between these gray bands, there is a domain in which *PI* was larger than 0.5. ***B***, Average firing rate of grcs and frequency of oscillatory spike generation. The average firing rate increased monotonically as the Mg^2+^ concentration increased. The oscillation frequency was around 7 Hz within the interval of 0.417–0.782 µM and around 20 Hz within the interval of 5.21–9.13 µM.

Figure 6***A*** shows the changes of the oscillation index *OI* and POT-representation index *PI* with Mg^2+^ concentration at *I*_MF_ = 22.7 pA. For 0.013 mM<[Mg^2+^]<0.260 mM, *PI* was almost constant at around 0.510. As Mg^2+^ concentration increased beyond 0.260 mM, *PI* decreased and reached 0.146 at [Mg^2+^] = 0.469 mM. Then, *PI* started to increase and reached a maximum at [Mg^2+^] = 1.40 mM. As Mg^2+^ concentration further increased, *PI* decreased again and vanished for [Mg^2+^]>6.52 mM. The POT-representing states emerged only in the interval of 0.782 mM<[Mg^2+^]<2.45 mM. *OI* was high in two separate domains for 0.417 mM<[Mg^2+^]<0.782 mM, and 5.21 mM<[Mg^2+^]<9.13 mM. Hence, the domain where the POT-representing state appeared was sandwiched by the two high-*OI* domains.

Figure 6***B*** shows the firing rate averaged over all grcs and the oscillation frequency. The average oscillation frequencies in the two high-*OI* domains were 7–8 Hz and 20–21 Hz. In contrast, the firing rate monotonically increased from 5 to 32 spikes/s as Mg^2+^ concentration increased.

### State transition controlled by the strength of an injected current

**Figure 7:**
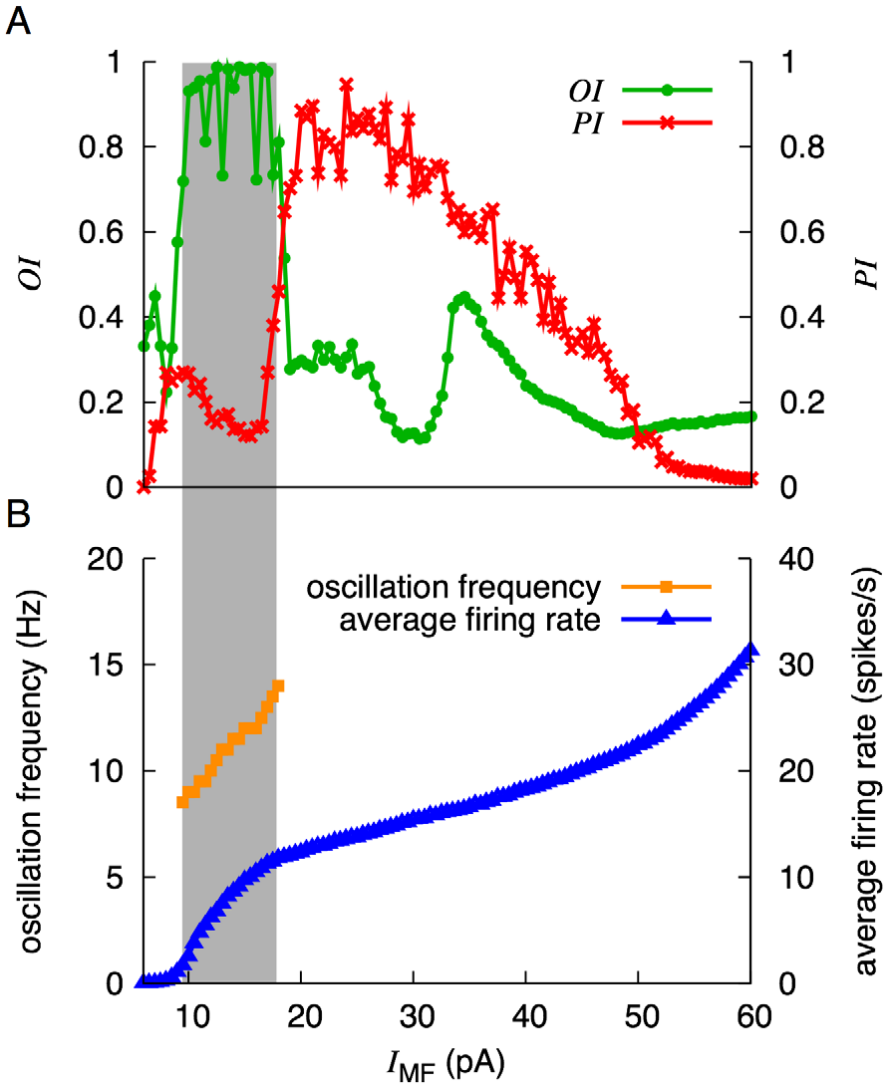
Dynamical states of the network for various amplitudes of the injected current at [Mg^2+^] = 1.2 mM. ***A***, *I*_MF_ dependence of *OI* and *PI*. High value of *OI* and *PI* tended to appear complementarily along the *I*_MF_ axis: *OI* was larger at a smaller current, whereas *PI* was larger at a larger current. ***B***, *I*_MF_ dependence of average firing rate and oscillation frequency of grcs. The average firing rate increased monotonically as *I*_MF_ increased. The oscillation frequency linearly increased from 9 to 14 Hz as *I*_MF_ increased from 9.5 to 18 pA within the high *OI* domain.

Next, we analyzed the changes of activity patterns as we varied continuously the strength of the injected current to grcs, *I*_MF_. Figure 7***A*** shows the changes of the oscillation index *OI* and POT-representation index *PI* with the strength of the injected current at [Mg^2+^] = 1.2 mM. For *I*_MF_<6.5 pA, it was too small for the grcs to be activated, and hence *PI* could not be defined. For 6.5 pA*<I*_MF_<16.5 pA, there was a local maximum of *PI* (*PI* = 0.269) at *I*_MF_ = 8.0 pA. *PI* exhibited a maximum at around *I*_MF_ = 26.5 pA. For *I*_MF_>26.5 pA, *PI* gradually decreased as *I*_MF_ increased. POT-representing states were well defined for 19 pA<*I*_MF_<33 pA. On the other hand, synchronized oscillation states were well defined in the interval of 9.5 pA<*I*_MF_<18 pA, whereas *OI* remained relatively small outside this interval.

Figure 7***B*** shows the average firing rate and the oscillation frequency of grcs against the strength of the injected current. The oscillation frequency is shown only in the range of the injected current in which oscillation states were well defined. The oscillation frequency was dissociated from the average firing rate. As *I*_MF_ increased up to *I*_MF_ = 60 pA, the firing rate monotonically increased from 0 to 31 spikes/s.

### State map in the parameter space

**Figure 8:**
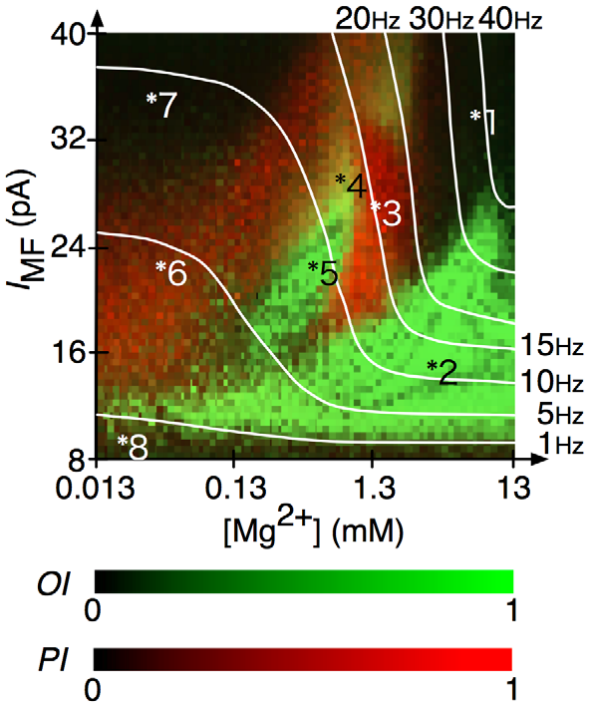
*OI* and *PI* for the present model in the space spanned by [Mg^2+^] and *I*_MF_. The abscissa and ordinate represent [Mg^2+^] and *I*_MF_, respectively. Values of *OI* are shown in the brightness of the green color, where the darkest shade indicates *OI* = 0 and brightest shade indicates *OI* = 1. Values of *PI* are shown by the brightness of the red color such that the darkest shade indicates *PI* = 0 and the brightest shade indicates *PI* = 1. Greenish or reddish domains indicate that the network exhibited synchronized oscillatory states or POT-representing states, respectively. Eight typical points are picked out from this parameter space. The white lines represent average firing rates of grcs of 1, 5, 10, 15, 20, 30 and 40 Hz.

**Figure 9:**
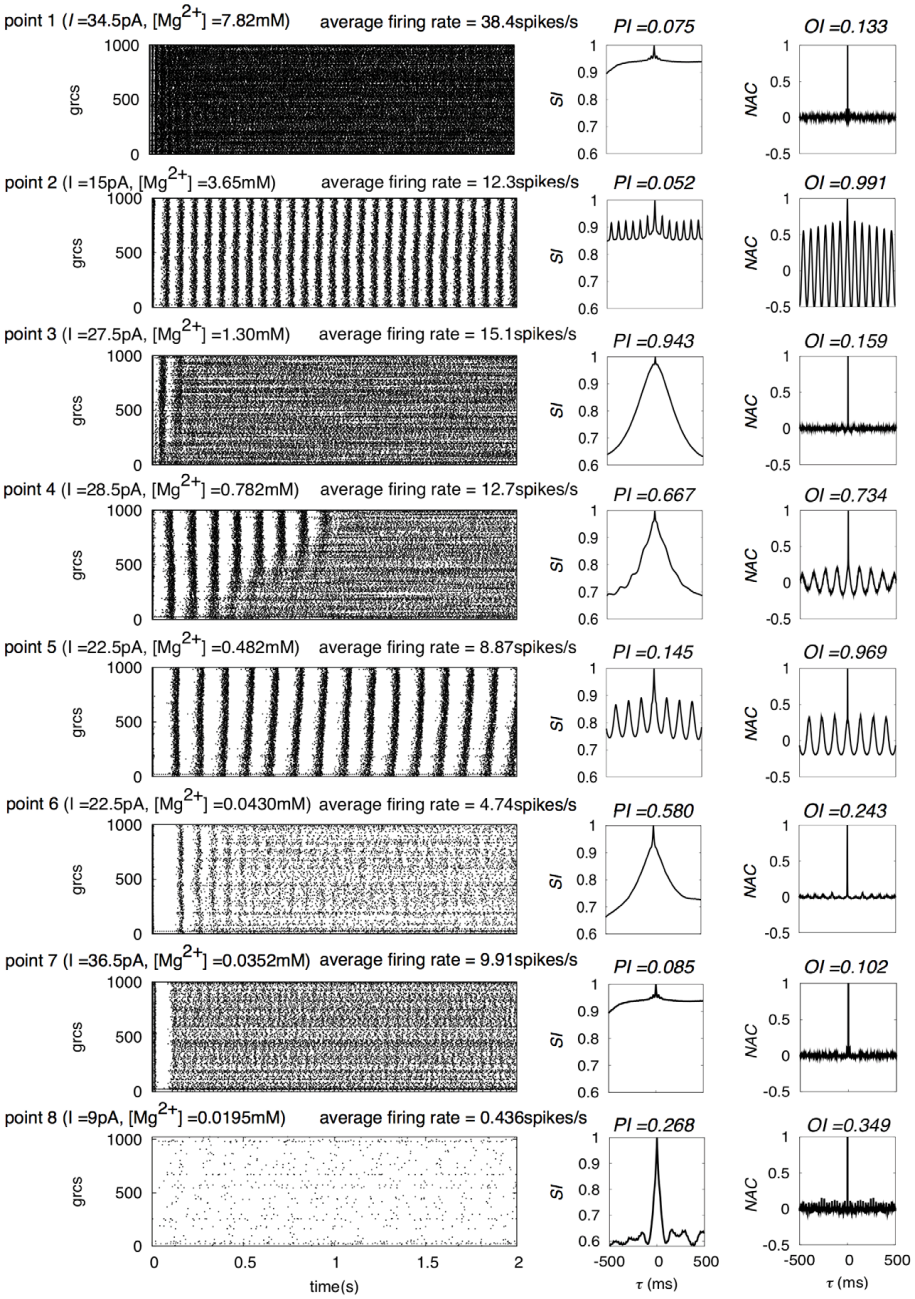
Spike patterns, similarity indices and normalized autocorrelation functions at eight points as in **Fig. 8**. The average firing rate, *PI* and *OI* are each figure given for each point.

Here, we explore all possible dynamical states in the present network by varying [Mg^2+^] and *I*_MF_ exhaustively. Figure 8 shows the values of oscillation index *OI* (green) and POT-representation index *PI* (red) of grc spike patterns (see also Figs. S2 ***A*** and ***B***). In Figure 9, we show spike trains, similarity indices and normalized autocorrelation functions of the network dynamics at eight representative points marked by asterisks in the state map shown in Fig. 8. Point 1 is characterized by an extremely high firing rate of grcs without oscillations. Point 1 corresponds to the case of a strong blockade of NMDA channels on the Goc dendrites. Because of the weaker inhibition of grcs by Gocs than the excitation of grcs by the injected current, both *PI* and *OI* had small values (*PI* = 0.075 and *OI* = 0.133), while the average firing rate was high (38.4 spikes/s). Point 2 exhibits synchronized oscillation induced by a small injected current. *PI*, *OI* and the oscillation frequency at point 2 were 0.052, 0.991 and 13 Hz, respectively. Points 1 and 2 are irrelevant to the POT representation. Points 3 and 6 are relevant to the POT representation. In particular, point 3 gives a typical POT-representing state, at which *PI* and *OI* were 0.943 and 0.159, respectively. Also at point 6, the grcs showed a POT-representing state due to the sustained opening of the NMDA channels at a low Mg^2+^ concentration. However, *PI* was lower than that at point 3 (*PI* = 0.580 and *OI* = 0.243). Point 4 was located at the transition from a synchronized oscillation state to a POT-representing state, because synchronized oscillation appeared transiently at the beginning and changed to a POT-representing state. As *I*_MF_ decreased, this transient synchronized oscillation became persistent as observed at point 5 (oscillation frequency  = 7 Hz; *OI* = 0.969), which was caused by the repeated bumping of strong excitation of Gocs mediated by NMDA channels. The lower firing rate of the grcs at point 7 was caused by strong inhibition from the Gocs, which exceeded the excitation by a large current injected to the grcs. Finally, point 8 corresponds to an almost inactive state of the grcs exhibiting neither a synchronized oscillation state nor a POT-representing state, because the injected current is too small and Gocs activated by persistently open NMDA channels inhibit the grcs strongly.

We classified the eight points into four different states: a synchronized oscillation state (State I), POT-representing state (State II), a metastable state (State III) and a uniform firing state (State IV). State I to IV are represented by the dynamical states at points 2 and 5, points 3 and 6, point 4 and points 1, 7 and 8, respectively. State II is observed when the average firing rate of the grcs is approximately 1–20 Hz (Fig. 8***B***). When Mg^2+^ concentration was 1.0–1.5 mM and *I*_MF_ was 22–33 pA, *PI* was higher and the average firing rate of grcs was almost constant. This shows that excitation and inhibition to grcs are balanced in this parameter range.

### Simulation of delay eyelid conditioning

Finally, we conducted additional simulations to confirm that the POT-representing states identified in this study can work to encode inter-stimulus intervals (ISIs) and a Purkinje cell (PC) can read out the intervals to elicit conditioned responses (CRs). We embedded a model PC in the granular layer network so that the PC received excitatory inputs from all grcs.

**Figure 10:**
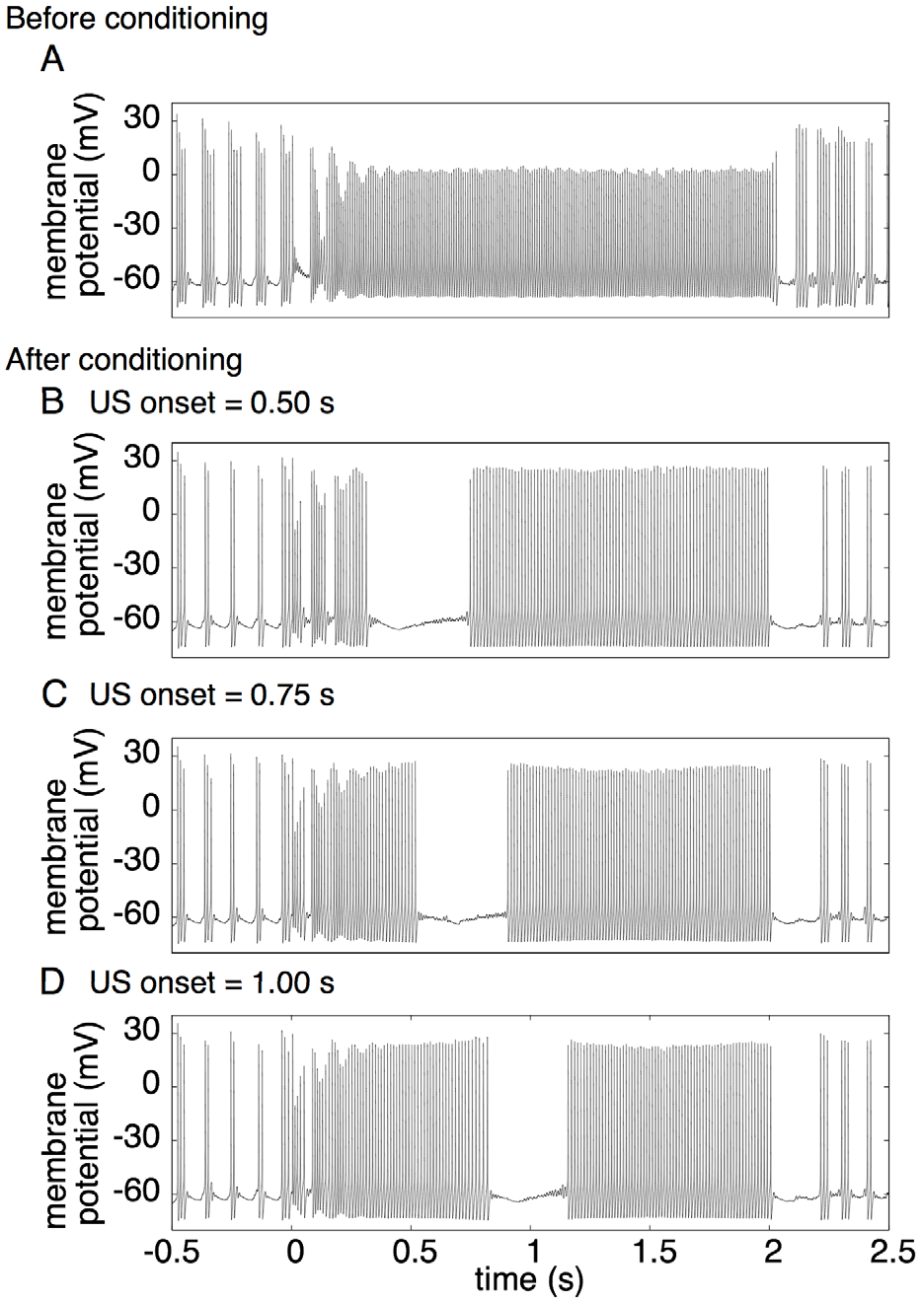
Membrane potential of a model PC in the simulation of delay eyelid conditioning. ***A***, Time evolution of membrane potential before conditioning. The abscissa indicates time from the CS onset. ***B***, ***C*** and ***D***, Time evolutions of membrane potential after conditioning in the cases where the US onset was set at 0.5, 0.75 and 1.0 s, respectively. The model PC learned to stop firing 100–200 ms earlier than the US onset for each conditioning.

Figure 10 shows the membrane potential of the model PC before and after the simulated conditioning. Before conditioning, the PC continuously elicited spikes at a high frequency during the CS presentation. In the simulated conditioning, the US signal was assumed to be given 0.75 s after the CS onset. The synaptic weights of the active PFs connected to the PC were set to zero 0.05–0.1 s before the US onset, whereas the synaptic weights of the other PFs were unchanged. After the conditioning, the model PC stopped firing about 200 ms earlier than the US onset and restarted at about 0.91 s. This result indicates that the model PC is able to learn the ISI between the CS and US onsets, which reproduces the experimental result of Jirenhed et al. [27]. We also confirmed that the model PC was able to learn other ISIs by setting the ISI to 0.5 or 1 s (Fig. 10). Moreover, we confirmed that other LTD time windows reported by Chen and Thompson [28] and Wang et al. [29] gave the same result (data not shown). When the CS signal was not presented, the model PC elicited burst spikes rhythmically at 9 Hz.

## Discussion

**Figure 11:**
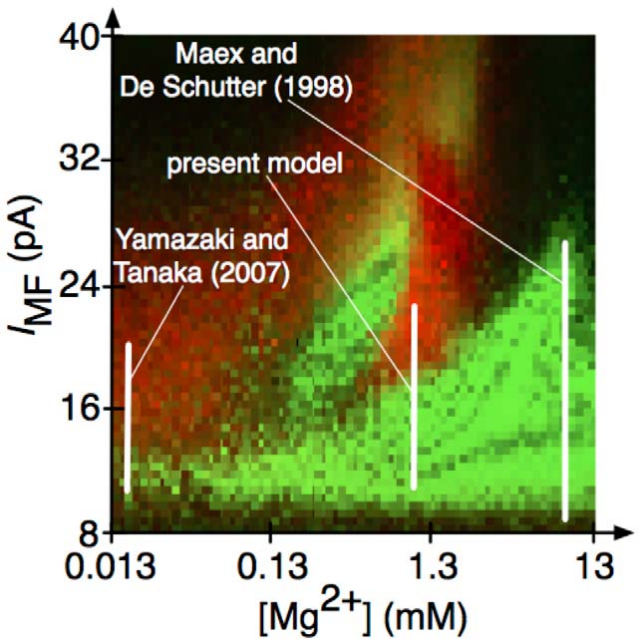
Parameter ranges mainly investigated in the present and previous research superimposed on the state map.

In the present study, we demonstrated that an identical computational model of the cerebellar granular layer can generate both synchronized oscillation states [7] and POT-representing states [3], [30]–[32] depending on the strength of a current injected to grcs (Fig. 11).When the network was driven by a small injected current which was assumed to represent spontaneous MF spike input, the model grcs and Gocs underwent synchronized oscillation at 9 Hz. This synchronized oscillation may be involved in the oscillation of LFP found in animals staying at rest [4], [5]. On the other hand, when a large current was injected to grcs, which was assumed to a CS signal fed to the network through MFs, the model grcs exhibited random burst-silent alternation of grc activities, which enables POT representation by active grc populations [3].

### Origin of random burst-silent alternation

We have shown that a POT can be represented by populations of active grcs, each of which exhibits random alternation of burst and silent modes. This raises a question of what the random burst-silent alternation originates in. Maex and De Schutter [7], in which synaptic weights of any connections in their network were random, did not show the random burst-silent alternation of grc activities. On the contrary, Yamazaki and Tanaka [3], in which the synaptic weights were constant though the connection pattern was random, demonstrated that the random alternation emerged at grcs. Simulations using a modified model in which grcs and Gocs were all connected with each other without connection randomness resulted in only coherent synchronized oscillations of grc and Goc activities. These observations indicate that randomness in synaptic connections between grcs and Gocs is more important than random fluctuation in synaptic weights of these connections for the generation of the random alternation. For the randomness in the external input, Medina et al. [31] and Yamazaki and Tanaka [3] assumed that randomly generated spikes were fed to model grcs through MFs in order to demonstrate that a POT can be represented by populations of active grcs. However, the present model with a constant current injection to grcs showed that the random burst-silent alternation does not require noisy input to grcs. Taken together, it is concluded that randomness in the connectivity between grcs and Gocs is a major cause of generating the random burst-silent alternation in grcs and Gocs.

### Influence of Golgi cells on the network dynamics

Buonomano and Mauk [32] have shown that the precision of a timed CR elicitation became worse with increase in weights of MF synapses on Gocs, whereas activity patterns of grcs became more resistant to noise in MF input signals. In addition, Maex and De Schutter [7] have observed that the synchronization of grc and Goc activities disappeared at stronger MF synapses on Gocs. These studies imply that the POT-representing states and synchronized oscillation states may disappear when we take into account direct MF inputs to Gocs, which are omitted in the present model. To examine the effects of the MF inputs to Gocs on the dynamical states, we performed additional simulations changing the ratio in strength of the current injected to Gocs to that injected to grcs, which would reflect the weight of MF synapses on Gocs relative to that on grcs, for two cases of a small and large currents injected to grcs. We found that *OI* was predominant over *PI* for any ratio of injected currents between 0 and 2 when the small current (10.7 pA) was injected to grcs, although both indices took small values when the ratio was large (Fig. S3 ***A***). For the large current injection (22.7 pA), *PI* was larger than *OI* when the ratio of injected currents was smaller than 0.3, whereas *OI* was larger than *PI* when the ratio was between 0.3 and 1.3 (Fig. S3 ***B***). The additional simulations by assuming a current injection to Gocs as well as grcs showed that there was a finite range of the strength of a current injected to Gocs, in which the POT-representing states or synchronized oscillation states appeared vigorously (Figs. S3 ***A*** and ***B***). These results are consistent with Buonomano and Mauk [32] for POT-representing states and Maex and De Schutter [7] for synchronized oscillation states. Therefore, the dynamical features of the present model are validated for weak MF inputs to Gocs.

Recently, Dugué et al. [33] have reported the existence of gap junctions between nearby Gocs and suggested that the oscillatory synchronization of Gocs can be induced from the electrical coupling. They also carried out computer simulation assuming the bath application of kainate to model Gocs and demonstrated that the Gocs mutually connected by gap junctions elicited spikes synchronously at 12 Hz. Their network model was composed of model Gocs alone. It is unclear whether the Goc synchronization is preserved when connections with grcs that receive spontaneous spike input through MFs are taken into account.

### Role of voltage dependence of NMDA channels in state transition

The importance of the voltage dependence of the NMDA channels was confirmed by simulations under various concentrations of Mg^2+^, which controls the opening of NMDA channels. We observed that the POT-representing states were disrupted by the blockade of NMDA channels increasing Mg^2+^ concentration, whereas the synchronized oscillation states were destabilized by the persistent opening of NMDA channels at a low Mg^2+^ concentration. Therefore, the NMDA channels are likely to be involved in the emergence of both the POT-representing states and the synchronized oscillation states. When the network was driven by the small current injection to grcs, simulating spontaneous MF spikes fed to grcs, the activities of the grcs were relatively low. Consequently, the excitatory input signals to Gocs were weak and Goc activities were also relatively low. This made the NMDA channels on the Gocs to open less effectively due to their voltage dependence. Thus, the depolarization of Gocs was largely driven by an AMPAR-mediated synaptic current, which resulted in the network behavior similar to that of Maex and De Schutter [7]'s model. In contrast, when a large current was injected to grcs, simulating strong MF signal input, the NMDA channels on the Goc dendrites opened persistently because of vigorous excitatory input signals from the grcs. This led to the random burst-silent alternation that can represent a POT, consistent with Yamazaki and Tanaka [3]'s model. Although the NMDA channels continually opened due to low Mg^2+^ concentration, synchronized oscillation was also observed (Figs. 6, 8 and 9). Synchronization in grc and Goc activities may appear at delicate balance among randomness in connectivity, the strength of a current injected to grcs, and the voltage dependence of NMDA channels. This problem should be one of the targets of future studies.

When we assumed the existence of NMDA channels only on the Goc somas, the random burst-silent alternation was not found at grcs even in response to the large current injection (data not shown). Specifically, the grcs did not exhibit persistent silent periods. This was because AHP following spike generation at Gocs quickly closed once-opened NMDA channels on the somas and the Gocs did not elicit burst spikes to produce the persistent silent periods of grcs. To open the NMDA channels for a longer time, NMDA channels should be located on the Goc dendrites somewhat electrically isolated from the somas, as assumed in the present model.

### Predictions based on the model

Gocs are regarded as playing an essential role in temporal information processing by the cerebellar cortex [34]. The present model specifically emphasizes the importance of voltage-dependent NMDA channels on the Goc dendrites. This leads to a prediction that blocking NMDA channels only on Gocs impairs delay eyelid conditioning due to the disruption of POT representation. This prediction may be examined experimentally. NMDA channels on grcs consist of NR1, NR2A and NR2C subunits [35], [36], whereas NMDA channels on Gocs consist of NR1, NR2B and NR2D subunits [36]. It is quite likely that NR2B subunits, which are exclusively expressed on Gocs, are responsible for the NMDA current of a long time constant [17], [37] that is needed for the temporal integration of grc activities. Therefore, one may be able to observe the impairment of delay eyelid conditioning by the bath application of selective NR2B antagonists [17], [37].

## Supporting Information

Figure S1 Normalized autocorrelation functions (***A***, ***B***, ***E***, ***F***, ***I*** and ***J***) and similarity indices (***C***, ***D***, ***G***, ***H***, ***K*** and ***L***) of Goc spike activities at default, high, and low values of [Mg^2+^] (from top to bottom), respectively. The left column (***A***, ***C***, ***E***, ***G***, ***I*** and ***K***) was calculated from Goc spike activities when a small current was injected to the grcs, whereas the right column (***B***, ***D***, ***F***, ***H***, ***J*** and ***L***) was calculated when a large current was injected.
(TIFF)

Figure S2 Network behaviors in the parameter space spanned by [Mg^2+^] and *I*_MF_. The oscillation index (***A***) and POT-representation index (***B***), which characterize the network dynamics, are plotted in the parameter space with different colors. Figure 8 was obtained by overlapping ***A*** and ***B***.
(TIFF)

Figure S3 Oscillation index (*OI*) and POT-representation index (*PI*) for the injection of a small current (I_MF-grc_ = 10.7 pA in ***A***) and a large current (*I*_MF-grc_ = 22.7 pA in ***B***) while varying the ratio in strength of the current injected to Gocs to that injected to grcs. ***A***, In the case of a small current injection to grcs, as the ratio increased, *OI* sharply decreased from nearly 1 to 0.3, whereas *PI* also decreased from 0.2 to nearly 0. ***B***, In the case of a large current injection to grcs, as the ratio increased, *OI* showed a plateau between 0.5 and 1.2 of the ratio, whereas *PI* sharply decreased from 0.9 to 0.1.
(TIFF)

Table S1 Tabular description of the present network model.
(DOC)
