# Supplementary figures and images for: Stimulus-Dependent State Transition between Synchronized Oscillation and Randomly Repetitive Burst in a Model Cerebellar Granular Layer
Source: PLoS Comput Biol. 2011 Jul 14;7(7):e1002087. doi: 10.1371/journal.pcbi.1002087 (PMC3136428; doi:10.1371/journal.pcbi.1002087)

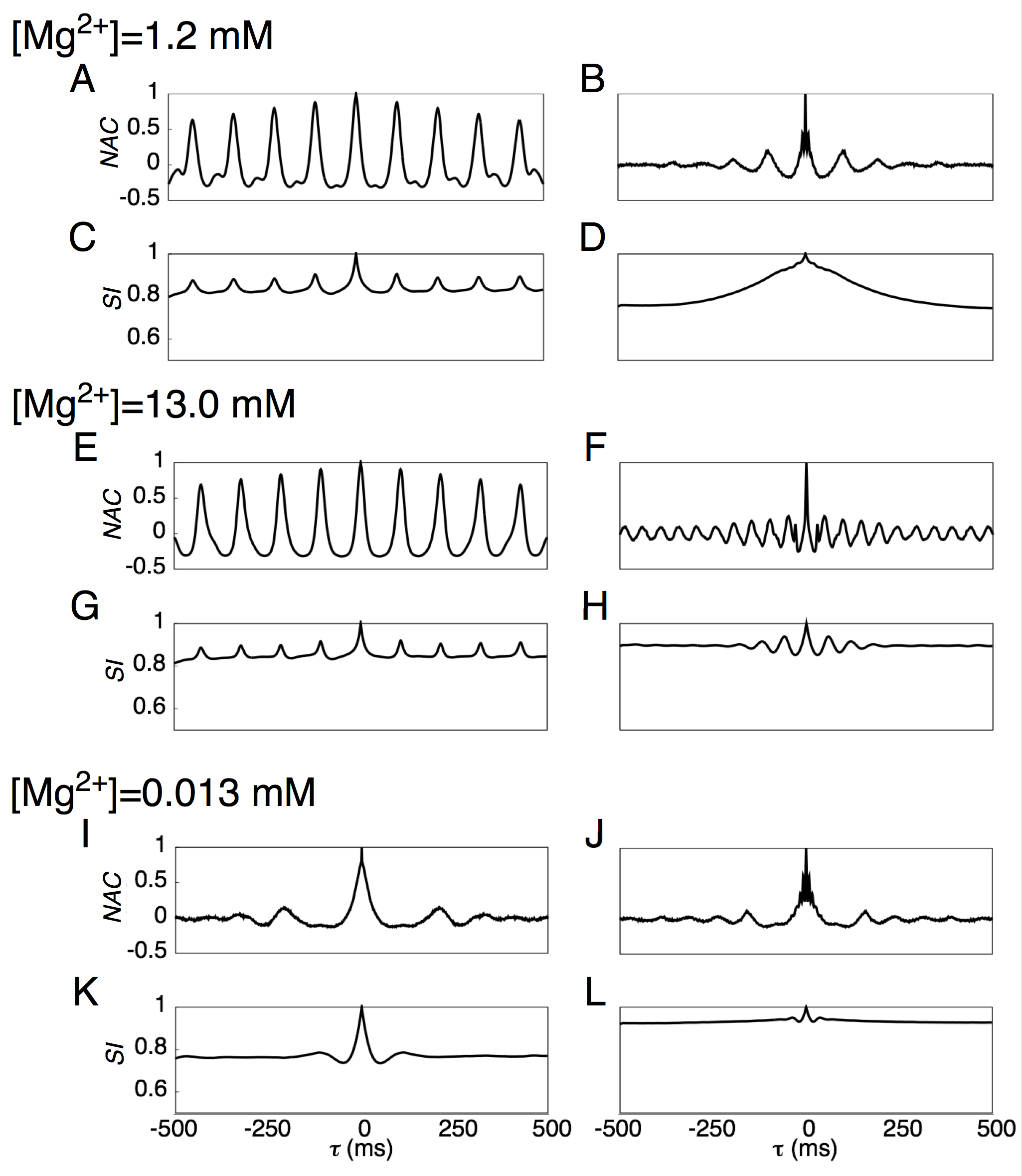

Supplement: Figure S1 — Normalized autocorrelation functions (A, B, E, F, I and J) and similarity indices (C, D, G, H, K and L) of Goc spike activities at default, high, and low values of [Mg2+] (from top to bottom), respectively. The left column (A, C, E, G, I and K) was calculated from Goc spike activities when a small current was injected to the grcs, whereas the right column (B, D, F, H, J and L) was calculated when a large current was injected. (TIFF) [file pcbi.1002087.s001.tiff]

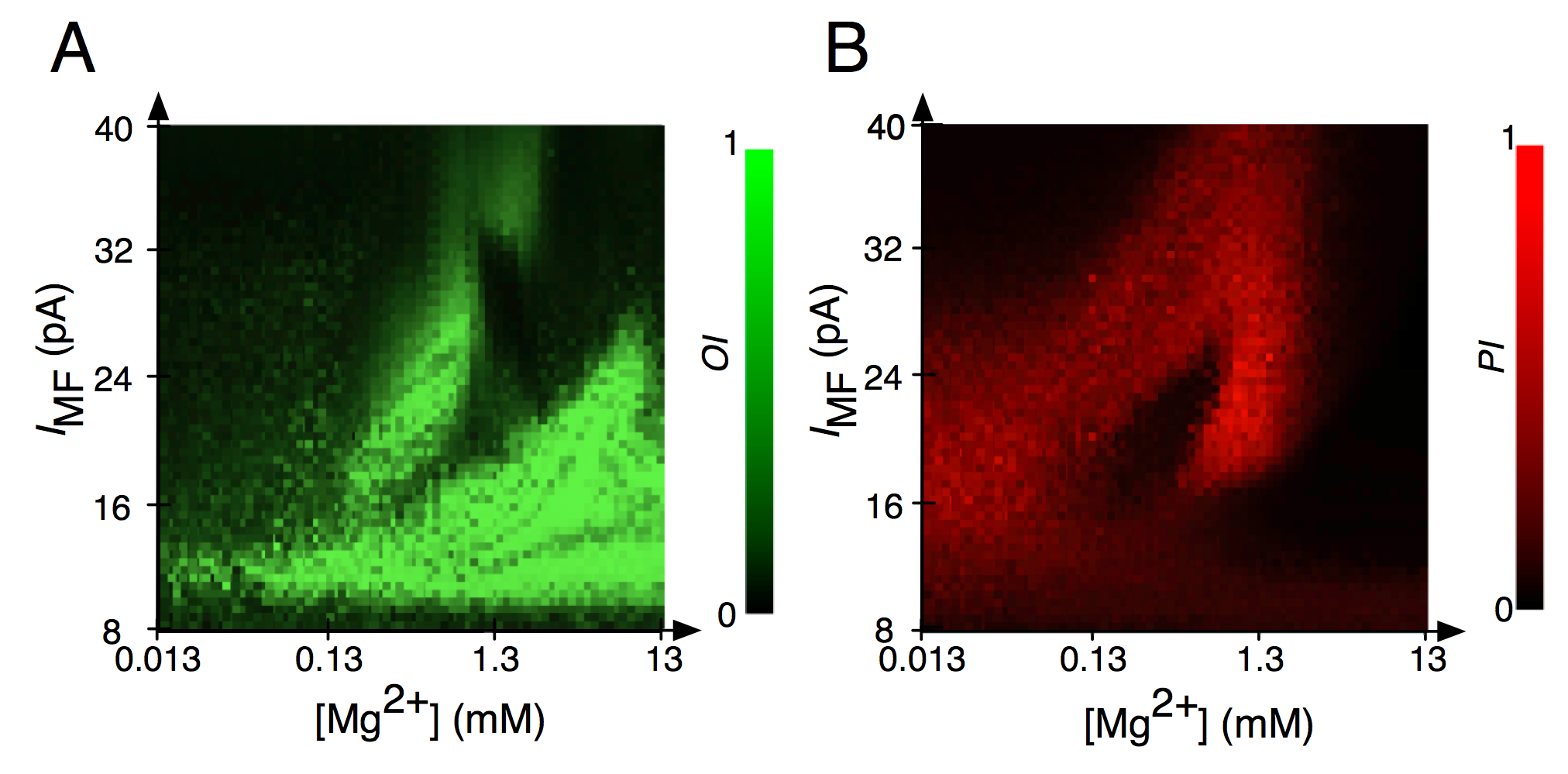

Supplement: Figure S2 — Network behaviors in the parameter space spanned by [Mg2+] and I MF. The oscillation index (A) and POT-representation index (B), which characterize the network dynamics, are plotted in the parameter space with different colors. Figure 8 was obtained by overlapping A and B. (TIFF) [file pcbi.1002087.s002.tiff]

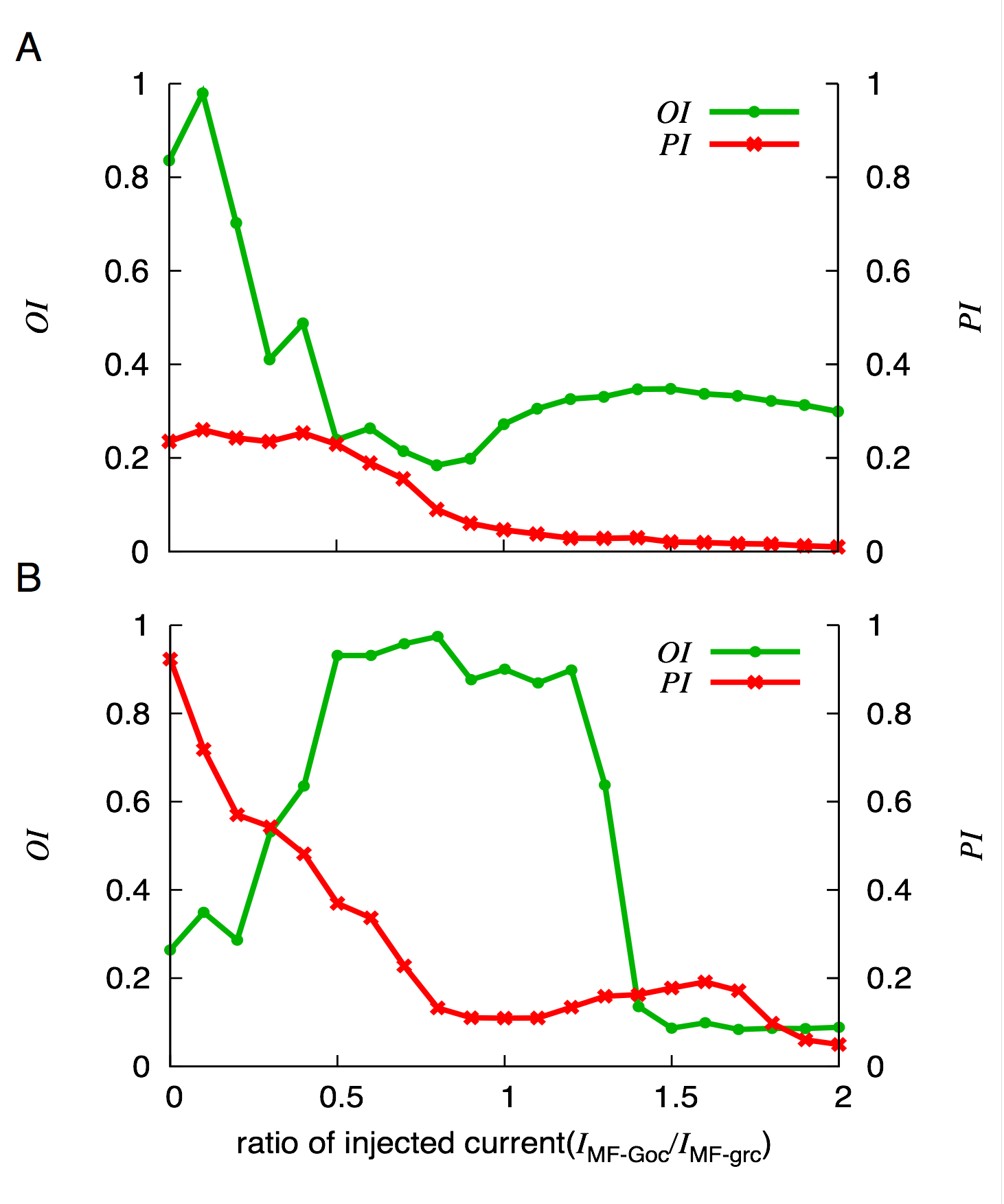

Supplement: Figure S3 — Oscillation index (OI) and POT-representation index (PI) for the injection of a small current (IMF-grc = 10.7 pA in A) and a large current (I MF-grc = 22.7 pA in B) while varying the ratio in strength of the current injected to Gocs to that injected to grcs. A, In the case of a small current injection to grcs, as the ratio increased, OI sharply decreased from nearly 1 to 0.3, whereas PI also decreased from 0.2 to nearly 0. B, In the case of a large current injection to grcs, as the ratio increased, OI showed a plateau between 0.5 and 1.2 of the ratio, whereas PI sharply decreased from 0.9 to 0.1. (TIFF) [file pcbi.1002087.s003.tiff]
