# Supplementary material for: Stimulus-Dependent State Transition between Synchronized Oscillation and Randomly Repetitive Burst in a Model Cerebellar Granular Layer
Source: PLoS Comput Biol. 2011 Jul 14;7(7):e1002087. doi: 10.1371/journal.pcbi.1002087 (PMC3136428; doi:10.1371/journal.pcbi.1002087)
Supplement: Table S1 — Tabular description of the present network model. (DOC) [file pcbi.1002087.s004.doc]

**Table S1 :** Tabular description of the present network model.

| Neuron Model | |
| --- | --- |
| Name | Granule cell　(grc) |
| Type | Hodgkin-Huxley |
| Dynamics | $I_{\text{applied}}=C\frac{dV}{dt}+\frac{1}{R}(V-V_{\text{leak}})+\sum_{\text{channel}} g_{\text{channel}}(V-V_{\text{channel}})$ $+\sum_{\text{synapse}} g_{\text{synapse}}(V-V_{\text{synapse}})+I_{\text{input}}$ with $C=3.14\text{pF,} R=\text{9.65G}\Omega, -70\text{mV}<V_{\text{leak}}<-60\text{mV}$ |
| Voltage-gated channels | $g_{\text{channel}}(t,V)=\bar{g}m^{P}h$ $\frac{dm}{dt}=\alpha(1-m)-\beta m, \tau=\frac{1}{\alpha+\beta}, m_{\infty}=\frac{\alpha}{\tau}$ Exponential(Exp): $\alpha(V)=Ae^{B(V_{m}-V_{0})}+C$ Sigmoid(Sig): $\alpha(V)=\frac{A}{1+e^{B(V_{m}-V_{0})}}$ Linoid(Lin): $\alpha(V)=\frac{A(V_{m}-V_{0})}{e^{B(V_{m}-V_{0})}-1}$ KCα: $\alpha(V,[\text{Ca}^{2+}]_{i})=\frac{A}{1+\frac{Ce^{BV_{m}}}{[\text{Ca}^{2+}]_{i}}}$ KCβ: $\alpha(V,[\text{Ca}^{2+}]_{i})=\frac{A}{1+\frac{[\text{Ca}^{2+}]_{i}}{Ce^{BV_{m}}}}$ |
| $\text{GABA}_{\text{A}}$ | $g_{\text{synapse}}(t,V)=\bar{g}\frac{(\tau_{1}-\tau_{2})(e^{-\frac{t}{\tau_{1}}}-e^{-\frac{t}{\tau_{2}}})}{\tau_{1}\tau_{2}(e^{-\frac{t_{\text{peak}}}{\tau_{1}}}-e^{-\frac{t_{\text{peak}}}{\tau_{2}}})}$ with $t_{\text{peak}}=\frac{\tau_{1}\tau_{2}\ln(\frac{\tau_{1}}{\tau_{2}})}{\tau_{1}-\tau_{2}}, t_{1}=100\text{ms}, t_{2}=5\text{ms}, \bar{g}=314\text{pS,}$ $V_{\text{synapse}}=-70\text{mV}$ |
| Calcium pools | $\frac{d[\text{Ca}^{2+}]_{i}}{dt}=\frac{I_{\text{CaL}}}{2FAd}-\frac{[\text{Ca}^{2+}]_{i}-[\text{Ca}^{2+}]_{\text{rest}}}{\tau}$ with $[\text{Ca}^{2+}]_{\text{rest}}=75.5\text{nM,} \text{F}=\text{96,494C/mol,}$ $\tau=\text{10ms,} \text{A}=314\mu\text{m}^{2}, d=0.084\mu\text{m}$ |
| Mossy fiber input | We regarded mossy fiber input as current injected to all grcs. |
| Golgi cell input | Each model grc randomly receives connections from Gocs within grc dendritic arborization diameter $315\text{μm}$(the mean and standard deviation of the connection is 6.11 and 2.57) (Fig.1). |

| Neuron Model | |
| --- | --- |
| Name | Golgi cell (Goc) |
| Type | Hodgkin-Huxley |
| Dynamics | $I_{\text{applied}}=C\frac{dV}{dt}+\frac{1}{R}(V-V_{\text{leak}})+\sum_{\text{channel}} g_{\text{channel}}(V-V_{\text{channel}})$ with $R_{\text{a}}=3.82\text{M}\Omega\text{,} -60\text{mV}<V_{\text{leak}}<-50\text{mV}$ |
| Voltage-gated channels | $g_{\text{channel}}(t,V)=\bar{g}m^{P}h$ $\frac{dm}{dt}=\alpha(1-m)-\beta m, \tau=\frac{1}{\alpha+\beta}, m_{\infty}=\frac{\alpha}{\tau}$ Exponential(Exp): $\alpha(V)=Ae^{B(V_{m}-V_{0})}+C$ Sigmoid(Sig): $\alpha(V)=\frac{A}{1+e^{B(V_{m}-V_{0})}}$ Linoid(Lin): $\alpha(V)=\frac{A(V_{m}-V_{0})}{e^{B(V_{m}-V_{0})}-1}$ KCα: $\alpha(V,[\text{Ca}^{2+}]_{i})=\frac{A}{1+\frac{Ce^{BV_{m}}}{[\text{Ca}^{2+}]_{i}}}$ KCβ: $\alpha(V,[\text{Ca}^{2+}]_{i})=\frac{A}{1+\frac{[\text{Ca}^{2+}]_{i}}{Ce^{BV_{m}}}}$ |
| $\text{AMPA}$ | $g_{\text{synapse}}(t,V)=\bar{g}\frac{(\tau_{1}-\tau_{2})(e^{-\frac{t}{\tau_{1}}}-e^{-\frac{t}{\tau_{2}}})}{\tau_{1}\tau_{2}(e^{-\frac{t_{\text{peak}}}{\tau_{1}}}-e^{-\frac{t_{\text{peak}}}{\tau_{2}}})}$ with $t_{\text{peak}}=\frac{\tau_{1}\tau_{2}\ln(\frac{\tau_{1}}{\tau_{2}})}{\tau_{1}-\tau_{2}}, t_{1}=0.5\text{ms}, t_{2}=0.03\text{ms}, \bar{g}=23,500\text{pS,}$ $V_{\text{synapse}}=0\text{mV}$ |
| $\text{NMDA}$ | $g_{\text{synapse}}(t,V)=\bar{g}\frac{(\tau_{1}-\tau_{2})(e^{-\frac{t}{\tau_{1}}}-e^{-\frac{t}{\tau_{2}}})}{\tau_{1}\tau_{2}(e^{-\frac{t_{\text{peak}}}{\tau_{1}}}-e^{-\frac{t_{\text{peak}}}{\tau_{2}}})(1+\eta[\text{Mg}^{2+}]e^{-\gamma V_{m}})}$ with $t_{\text{peak}}=\frac{\tau_{1}\tau_{2}\ln(\frac{\tau_{1}}{\tau_{2}})}{\tau_{1}-\tau_{2}}, t_{1}=100, t_{2}=5, \bar{g}=2,520\text{pS,}$ $V_{\text{synapse}}=0\text{mV,} \eta=0.00092\text{mM}^{\text{-1}}, \gamma=150\text{mV}^{\text{-1}}, [\text{Mg}^{2+}]=1.2\text{mM}$ |
| Calcium pools | $\frac{d[\text{Ca}^{2+}]_{i}}{dt}=\frac{I_{\text{CaL}}}{2FAd}-\frac{[\text{Ca}^{2+}]_{i}-[\text{Ca}^{2+}]_{\text{rest}}}{\tau}$ with $[\text{Ca}^{2+}]_{\text{rest}}=75.5\text{nM,} \text{F}=\text{96,494C/mol,}$ $\tau=\text{200ms,} \text{A}=2,826\mu\text{m}^{2}, d=0.091\mu\text{m}$ |
| granule cell input | Each model Goc randomly connected from closed $9\times32$PFs (the mean and standard deviation of the connection is 26.80 and 6.50) (Fig.1). |

| Parameter values of voltage-gated channels for model grc and Goc | | | | | | | | | | | |
| --- | --- | --- | --- | --- | --- | --- | --- | --- | --- | --- | --- |
| Channel | $V_{\text{channel}}$(mV) | *P* |  |  |  | A  (ms^-1^) | B  (ms^-1^) | C  (ms^-1^) | $V_{0}$ (mV) | Constraints | $\bar{g}$ (nS) |
| NaF | 55 | 3 | m | $\alpha$ | Exp | 7.5 | 0.081 |  | -39 |  | grc  172  Goc  1,131 |
|  |  |  |  | $\beta$ | Exp | 7.5 | -0.066 |  | -39 | $\tau>0.01 \text{ms}$ |
|  |  |  | h | $\alpha$ | Exp | 0.6 | -0.089 |  | -50 |  |
|  |  |  |  | $\beta$ | Exp | 0.6 | 0.089 |  | -50 | $\tau>0.045 \text{ms}$ |
| Kdr | -90 | 4 | m | $\alpha$ | Exp | 0.85 | 0.073 |  | -38 |  | grc |
|  |  |  |  | $\beta$ | Exp | 0.85 | -0.018 |  | -38 |  | 28  Goc  192 |
|  |  |  | h | $\alpha$ | Exp | 3e-4 | -0.08 | 35e-4 | -46 | $\alpha>\text{38e-4}$ |
|  |  |  |  | $\beta$ | Sig | 5.5e-3 | -0.0807 |  | -44 |  |
| CaL | 80 | 2 | m | $\alpha$ | Sig | 8.0 | -0.072 |  | 5 |  | grc  2.9  Goc  23.5 |
|  |  |  |  | $\beta$ | Lin | 0.1 | 0.2 |  | -8.9 |  |
|  |  |  | h | $\alpha$ | Exp | 0.025 | -0.05 |  | -60 | $\alpha>0.025$ |
|  |  |  |  | $\beta$ | Exp | -0.025 | -0.05 | 0.025 | -60 | $\beta>0.0$ |
| H | -42 | 1 | m | $\alpha$ | Exp | 4e-3 | -0.0909 |  | -75 |  | grc 97.1  Goc 4.85 |
|  |  |  |  | $\beta$ | Exp | 4e-3 | 0.0909 |  | -75 |  |
| KA | -90 | 3 | m | $\tau$ | Exp | 0.41 | 1/42.8 | 0.167 | -43.5 |  | grc  3.6  Goc  14.8 |
|  |  |  |  | $\text{m}_{\infty}$ | Sig | 1 | -1/19.8 |  | -46.7 |  |
|  |  |  | h | $\tau$ | $\tau(V)=10.8+0.03V_{\text{m}}+1/(57.9e^{0.127V_{\text{m}}}+0.000134e^{-0.059V_{\text{m}}})$ | | | | | |
|  |  |  |  | $\text{h}_{\infty}$ | Sig | 1 | 1/8.4 |  | -78.8 |  |
| KC | -90 | 1 | m | $\alpha$ | $\text{KC}\alpha$ | 12.5 | -0.085 | 1.5e-3 |  |  | grc 56.5  Goc 16.2 |
|  |  |  |  | $\beta$ | $\text{KC}\beta$ | 7.5 | -0.077 | 150e-6 |  |  |

| Neuron Model | |
| --- | --- |
| Name | Purkinje cell　(PC) |
| Type | Hodgkin-Huxley |
| Dynamics | $I_{\text{applied}}=C\frac{dV}{dt}+\frac{1}{R}(V-V_{\text{leak}})+\sum_{\text{channel}} g_{\text{channel}}(V-V_{\text{channel}})$ $+\sum_{\text{synapse}} g_{\text{synapse}}(V-V_{\text{synapse}})$ with $C=1.0\text{nF,} R=\text{0.3G}\Omega, V_{\text{leak}}=-60\text{mV}$ |
| Voltage-gated channels | $g_{\text{channel}}(t,V)=\bar{g}m^{P}h$ $\frac{dm}{dt}=\alpha(1-m)-\beta m, \tau=\frac{1}{\alpha+\beta}, m_{\infty}=\frac{\alpha}{\tau}$ Exponential(Exp): $\alpha(V)=Ae^{B(V_{m}-V_{0})}+C$ Sigmoid(Sig): $\alpha(V)=\frac{A}{1+e^{B(V_{m}-V_{0})}}$ Linoid(Lin): $\alpha(V)=\frac{A(V_{m}-V_{0})}{e^{B(V_{m}-V_{0})}-1}$ |
| $\text{AMPA}$ | with $\tau_{1}=30\text{ms,} \bar{g}=105\text{mS}, V_{\text{synapse}}=5\text{mV}$ |
| Parallel fiber input | Model PC connected with all grcs. |

| Parameter values of voltage-gated channels for model PC | | | | | | | | | | | |
| --- | --- | --- | --- | --- | --- | --- | --- | --- | --- | --- | --- |
| Channel | $V_{\text{channel}}$(mV) | *P* |  |  |  | A  (ms^-1^) | B  (ms^-1^) | C  (ms^-1^) | $V_{0}$ (mV) | Constraints | $\bar{g}$ (nS) |
| Na^+^ | 55 | 3 | m | $\alpha$ | Lin | 1.0 | -0.1 |  | 35 |  | 120 |
|  |  |  |  | $\beta$ | Exp | 4.0 | -1/18 |  | 60 |  |
|  |  |  | h | $\alpha$ | Exp | 0.07 | -0.05 |  | 60 |  |
|  |  |  |  | $\beta$ | Sig | 1.0 | -0.1 |  | 30 |  |
| K^+^ | -72 | 3 | m | $\alpha$ | Lin | -0.01 | -0.01 |  | 50 |  |  |
|  |  |  |  | $\beta$ | Exp | 0.125 | -0.0125 |  | 60 |  | 36 |
|  |  |  | h | $\alpha$ | Lin | -0.01 | -0.01 |  | 50 |  |
|  |  |  |  | $\beta$ | Exp | 0.125 | -0.0125 |  | 60 |  |
